# Planktonic Bacterial and Archaeal Communities in an Artificially Irrigated Estuarine Wetland: Diversity, Distribution, and Responses to Environmental Parameters

**DOI:** 10.3390/microorganisms8020198

**Published:** 2020-01-31

**Authors:** Mingyue Li, Tiezhu Mi, Zhigang Yu, Manman Ma, Yu Zhen

**Affiliations:** 1 College of Environmental Science and Engineering, Ocean University of China, Qingdao 266100, China; 2 Laboratory for Marine Ecology and Environmental Science, Qingdao National Laboratory for Marine Science and Technology, Qingdao 266071, China; 3 Key Laboratory of Marine Environment and Ecology, Ministry of Education, Ocean University of China, Qingdao 266100, China; 4 Key Laboratory of Marine Chemistry Theory and Technology, Ministry of Education/Institute for Advanced Ocean Study, Ocean University of China, Qingdao 266100, China

**Keywords:** archaea, bacteria, microbial community, estuarine wetland, wetland effluent

## Abstract

Bacterial and archaeal communities play important roles in wetland ecosystems. Although the microbial communities in the soils and sediments of wetlands have been studied extensively, the comprehensive distributions of planktonic bacterial and archaeal communities and their responses to environmental variables in wetlands remain poorly understood. The present study investigated the spatiotemporal characteristics of the bacterial and archaeal communities in the water of an artificially irrigated estuarine wetland of the Liaohe River, China, explored whether the wetland effluent changed the bacterial and archaeal communities in the Liaohe River, and evaluated the driving environmental factors. Within the study, 16S rRNA quantitative PCR methods and MiSeq high-throughput sequencing were used. The bacterial and archaeal 16S rRNA gene abundances showed significant temporal variation. Meanwhile, the bacterial and archaeal structures showed temporal but not spatial variation in the wetland and did not change in the Liaohe River after wetland drainage. Moreover, the bacterial communities tended to have higher diversity in the wetland water in summer and in the scarce zone, while a relatively higher diversity of archaeal communities was found in autumn and in the intensive zone. DO, pH and PO_4_-P were proven to be the essential environmental parameters shaping the planktonic bacterial and archaeal community structures in the Liaohe River estuarine wetland (LEW). The LEW had a high potential for methanogenesis, which could be reflected by the composition of the microbial communities.

## 1. Introduction

Located at the transition zone of terrestrial and aquatic systems, wetlands include different types of environments that play various functions in ecosystems. Estuarine wetlands are particularly important ecosystems because of their large number, considerable size, various ecological functions, and distinct location advantages [1]. The processes of biogeochemical cycling, purification and energy transfer in estuarine wetlands cannot take place without the activities of microorganisms. Conversely, the diversity, composition and structure of microbial communities can also be affected by the environmental compatibility of wetlands [2,3,4]. In wetland systems, many kinds of microbes inhabit the water, sediments and plants and have different functions in response to the surrounding environment [5,6,7].

Some previous investigations have shown the diversity, composition and structure of microbial communities in estuarine wetlands [8,9] as well as the interactions between microbial communities and environmental variables (e.g., salinity, pH, nitrogen content, sulfur content, total organic carbon, iron and carbon and nitrogen mineralization) [7,8,9,10]. However, these studies have emphasized the soils, sediments and rhizospheres of plants in estuarine wetlands, and therefore, the microbial community in the water requires more illumination to better understand the whole estuarine wetland ecosystem. In addition, despite their potential importance in estuarine wetland ecosystems, there is still limited information on the temporal and spatial variation in the diversity, abundance, composition and structure of the microbial communities in estuarine ecosystems [9]; information is especially lacking regarding the locations where water discharges into estuarine systems, and whether this water transfer influences the microbial community in the receiving areas remains unknown.

The Liaohe River estuarine wetland (LEW) is dominated by *Phragmites australis* and *Suaeda heteroptera*, and tidal flats are successively distributed in the area [11]. Over the past few decades, intensive human activities, such as agricultural practices, aquaculture production, petrochemical industry development and urbanization, have significantly affected the nutrient biogeochemistry of the estuary [3]. The distribution of the microbial community in the LEW has been evaluated in previous studies; however, traditional molecular ecological methods have usually been used to carry out the research [10,12], and emphasis has been placed on the soil [13]. More studies on the microbial community in the LEW are required to compare this system with other estuarine wetland ecosystems.

Recently, the development of high-throughput sequencing has provided new insights into microbial communities at a molecular level and revolutionized our view of microbial diversity with molecular phylogenetic approaches [4,14]. Bioinformatics combined with statistical analysis have been widely used to analyze numerous and complex experimental data [2,4]. High-throughput sequencing and related analytical methods were applied in many studies to study microbial communities [2,4]. In this study, environmental parameter measurement, real-time PCR (qPCR), Illumina 16S ribosomal RNA (rRNA) amplicon sequencing and various statistical analyses were applied. We aimed (i) to investigate the spatiotemporal characteristics of the bacterial and archaeal communities in the wetland water; (ii) to identify the critical environmental parameters influencing the bacterial and archaeal communities in the LEW; and (iii) to determine whether wetland effluent changed the bacterial and archaeal communities in the Liaohe River, as the wetland effluent eventually discharges into the Liaohe River.

## 2. Materials and Methods

### 2.1. Study Area

**Figure 1:**
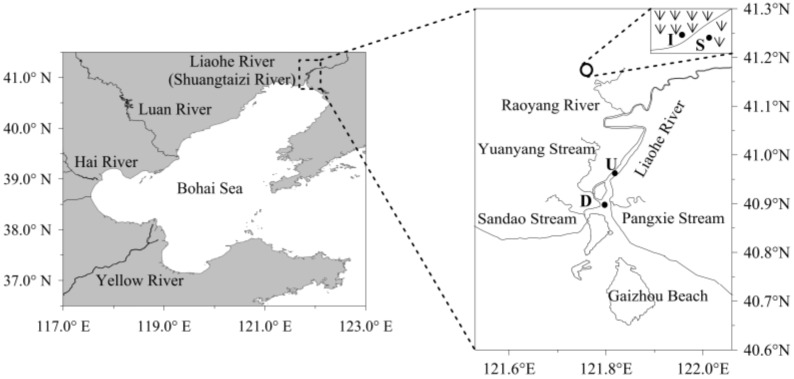
Location of the sampling station in the Liaohe River estuarine wetland (LEW). The uppercase letters S and I refer to the scarce and intensive reed zones in the wetland, respectively, while U and D refer to the upstream and downstream zones of the outlet from the wetland to the Liaohe River, respectively.

Located north of the Bohai Sea in northeastern China and situated in the warm temperate zone, which has a semihumid continental monsoon climate, the LEW (121°30′–122°00′ E, 40°45′–41°10′ N) encompasses a marsh area of approximately 1000 km^2^. The average annual temperature ranges from 8.3–8.4 °C. The average annual precipitation ranges from 611.6–640.1 mm, but the average annual evaporation ranges from 1392–1705 mm [15]. In recent years, because of scarce precipitation and the unreasonable utilization of water resources, the wetland has been artificially irrigated by water conservancy projects to improve reed production. We chose a *Phragmites australis* wetland as the study site (Figure 1). The wetland is artificially irrigated with water from the Liaohe River, and the water from the wetland is drained every autumn to harvest reeds and flows into the Liaohe River through the Yuanyanggou stream and then into the sea. Therefore, the upstream and downstream areas where the Yuanyanggou stream joins the Liaohe River were also investigated in this study (Figure 1).

### 2.2. Sample Collection and Environmental Parameter Analysis

The first and second sampling sites were in the scarce (S) and intensive (I) zones of the reed wetland, respectively. The third and fourth sites were upstream (U) and downstream (D) of the outlet of the wetland that flows into the Liaohe River, respectively (Figure 1). At the first and second sampling sites, samples were collected every six hours on 16th May, 15th June and 13th September in 2015, which were classified as the initial growth, rapid growth and mature stages of reeds, respectively. At the third and fourth sampling sites, samples were also collected every six hours on October 1st, when the water in the wetland was discharged into the Liaohe River to harvest the reeds. Each water sample was obtained using a bucket and poured into a 2 L sterile glass bottle for further treatments.

For the molecular experiment, approximately 300 mL water samples were filtered through 0.22 μm polycarbonate filters (47 mm, Whatman, Maidstone, UK). All samples were stored at −80 °C until DNA analysis. The temperature (T), pH, dissolved oxygen (DO), and salinity (SAL) of the water were determined in situ using an HQ40d multiparameter meter (HACH, Loveland, CO, USA). The water samples for the dissolved organic carbon (DOC) and nutrient analyses were filtered through 0.7 μm GF/F filters and 0.45 μm cellulose acetate filters and then surveyed with a TOC-VCPH analyzer (Shimadzu, Kyoto, Japan) and a QuAAtro nutrient autoanalyzer (Seal Analytical, Norderstedt, Germany), respectively. The determination of chlorophyll a (Chla) was performed by the acetone method after the water samples were filtered with 0.45 μm cellulose acetate filters. More detailed information regarding the environmental parameter analyses can be found in a previous study [16].

### 2.3. DNA Extraction and qPCR

The total genomic DNA of each water sample was extracted from filters. A filter for each sample was cut into pieces and added to the PowerBead Tubes provided by the PowerSoil Isolation Kit (MO Bio Laboratories, San Diego, CA, USA). The subsequent treatments were in accordance with the manufacturer’s instructions except that 60 μL of Solution C6 was added in the last step. The DNA products from the day and night samples (*n* = 4) were thoroughly mixed in equal amounts to make the results more realistic. The quality and quantity of the extracted DNA were verified with a NanoDrop^TM^ spectrophotometer (Thermo Scientific, Waltham, MA, USA) and agarose gel electrophoresis, respectively, and stored at −20 °C until use.

For the quantification of the bacterial and archaeal 16S rRNA genes, qPCR was performed using an Applied Biosystems^TM^ 7500 Real-Time PCR System (Life Technologies, Forster City, CA, USA) in triplicate with the fluorescent dye SYBR Green. The bacterial primers 338F (5′-ACTCCTACGGGAGGCAGCAG)/806R (5′-GGACTACHVGGGTWTCTAAT) [17] and the archaeal primers U519F (5′-CAGYMGCCRCGGKAAHACC)/806R (5′-GGACTACNSGGGTMTCTAAT) [18] were used to determine the copy numbers of the 16S rRNA genes. Melting curve analysis was performed to confirm the specificity of each amplicon.

### 2.4. Illumina 16S rRNA Gene Amplicon Sequencing

The 16S rRNA genes were amplified using bacterial primer set 343F (5′-TACGGRAGGCAGCAG)/798R (5′-AGGGTATCTAATCCT) [19] and archaeal set Arch344F (5′-ACGGGGYGCAGCAGGCGCGA)/Arch915R (5′-GTGCTCCCCCGCCAATTCCT) [20]. In the first amplification, the PCR mixture was composed of 15 μL of 2× Taq Master Mix, 1 μL of each primer, 50 ng of DNA template and diluted to a final volume of 30 μL with sterile water. The PCR conditions were 94 °C for 5 min, followed by 25 cycles consisting of 94 °C for 30 s, 56 °C for 30 s and 72 °C for 30 s, 2 °C for 7 min and ending at 4 °C. After purification, to obtain the barcoded sequences, 50 ng of the PCR products were added to the same 30 μL mixture for the second amplification and purification. The PCR conditions were the same as those used for the first amplification except that the number of cycles was six. An Illumina Sequencer MiSeq PE300 (San Diego, CA, USA) was used for the high-throughput sequencing by OE Biotechnology Company (Shanghai, China).

The obtained raw paired-end reads were trimmed using Trimmomatic software (v0.35) [21] before being assembled using FLASH software (v1.2.11) [22]. Then, the sequences were denoised and filtered to guarantee the quality of the reads using QIIME software (v8.0) [23]. Briefly, any reads with ambiguous, homologous or short (less than 200 bp) sequences were discarded, and the reads with 75% of the bases above Q20 were retained. Then, reads with chimeras were detected and removed. The remaining high-quality reads were clustered into operational taxonomic units (OTUs) using VSERCH (v2.4.2) with 97% similarity [24]. The representative read of each OTU was selected using the QIIME package and annotated and blasted against the Silva database (v123) [25] using the RDP classifier (v2.2) [26]. The data generated by high-throughput sequencing were deposited into the Sequence Read Archive (SRA) database under accession number SRP199281.

### 2.5. Data Analysis

Spearman’s rank correlation coefficients were used to assess correlations between two independent variables. Partial correlation analysis was added to determine the relationships between the environmental parameters and the detection frequencies of the dominant microbial taxa to remove autocorrelation between the environmental parameters. Mann–Whitney and Wilcoxon tests were used to assess differences between two independent groups in different conditions. Stepwise regression analysis was used to evaluate the effect of the environmental parameters on the α-diversity indices. SPASS v17.0 (SPASS, Inc., Chicago, IL, USA) was used for all analyses.

To obtain the distribution pattern of the LEW microbial community structure, detrended correspondence analysis (DCA) was used first to choose a linear or unimodal model. Then, correspondence analysis (CA) or principal components analysis (PCA) was performed. Accordingly, canonical correspondence analysis (CCA) was performed to verify the effects of the environmental factors on microbial community variation in the LEW. The significance of the effects was determined with a Monte Carlo permutation test (999 random unrestricted permutations). Prior to performing CCA, closely autocorrelated environmental parameters (*r* > 0.08, *p* < 0.05) were filtered, and the top environmental parameters that were correlated with microbial community structure were selected using BIOENV/BVSTEP. Furthermore, a Mantel test was also used to calculate the relationship between the environmental parameters and the microbial community structure. BIOENV/BVSTEP was performed with PRIMER 5 software, and the DCA, CA, PCA, CCA, Monte Carlo permutation test and Mantel test were performed using R v3.4.2 with the vegan package.

## 3. Results

### 3.1. Characterization of the Environmental Parameters

Based on a previous study [16], the averages (*n* = 4) of the environmental parameters from the LEW were calculated, which are listed in Table S1. The main characterization of the environmental parameters had been reported in a published study [16]; therefore, emphasis was placed on correlations between the environmental parameters to better understand the relationship between the microbial community and the environmental variables. Spearman’s correlation analysis indicated that T, SAL, dissolved inorganic nitrogen (DIN), NO_2_-N and the DIN/PO_4_-P ratio were highly significantly correlated with each other (*r* > |0.821|, *p* < 0.05) (Table S2), and DIN was also closely associated with NO_3_-N (*r* = 0.857, *p* = 0.014). In addition, DO was negatively related to total phosphorus (TP) and positively related to Chla (Table S2). In general, notable physical and chemical variations were found between the different months (Mann–Whitney test, *p* < 0.05), whereas no significant differences were observed between the S zone and I zone (Table S3). For example, the DO concentration was significantly higher in September than in May and June (Mann–Whitney test, *p* < 0.05), and the Chla content was significantly different only between May and September (Mann–Whitney test, *p* < 0.05). For the U and D areas, some of the nutrient concentrations and the salinity distinctly changed following the discharge of wetland water (Table S3).

### 3.2. Bacterial and Archaeal Community α-Diversity

For the bacteria, a total of 116,150 high-quality sequences were obtained from the studied water samples in the LEW. Each bacterial library had 14,777 to 17,842 reads and was normalized to 14,776 reads after being resampled randomly. The number of OTUs ranged from 192–379 (clustered at the 97% identity level), which were well captured and proven by the high Good’s coverage (≥99.35%, Table 1). The species diversity represented by the Shannon index ranged from 3.17 to 5.66. The highest diversity was found in June in the wetland water samples, followed by September and May, and the species diversity was higher in the D zone than in the U zone of the outlet water samples (Table 1). The richness represented by the Chao 1 index ranged from 259.00 to 493.38, and the evenness estimated by Pielou index ranged from 0.39 to 0.66 in all water samples (Table 1). The evenness showed the same regulator with the diversity, that evenness was highest in June and lowest in May, whereas richness showed a different pattern. Additionally, the diversity and evenness of the bacterial communities were higher in the S zone than in the I zone.

A total of 267,253 valid reads of archaea were obtained after quality control, ranging from 27,696 to 53,552 reads of each sample, with an average of 38,179 ± 8711 reads. A total of 69 to 805 OTUs were calculated after each archaeal library was normalized to 27,695 reads after being resampled randomly (Table 1). Different with the bacterial results, the highest Shannon diversity, Chao 1 richness and Pielou evenness values of the archaeal communities were observed in September in the wetland water samples, followed by June and May; the diversity and richness of the archaeal communities were higher in the I zone than in the S zone. Regarding the samples in the U and D zones of the outlet from the wetland to the Liaohe River, the archaeal community diversity, richness and evenness in the D sample were higher (Table 1). Overall, the α-diversity of the bacterial communities tended to be higher in the S zone and in June, while that of the archaeal communities tended to be higher in the I zone and in September. The discharge of wetland water increased the α-diversity of the bacterial and archaeal communities in the D water compared with those in the U water.

### 3.3. Bacterial and Archaeal Community Structures

**Figure 2:**
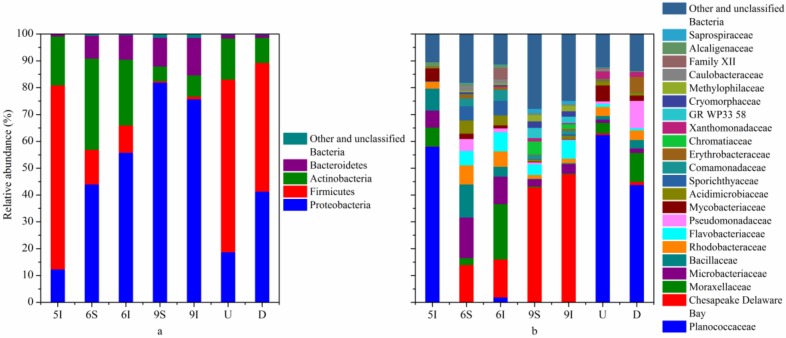
Taxonomic composition of the bacterial communities in the LEW. Rare bacterial populations accounted for <0.5% of the total sequences, and unclassified bacteria were included in the group “Other and unclassified Bacteria”. (**a**) Phylum and (**b**) family classification levels.

The taxonomic identification results showed that the high-quality reads of bacteria obtained from all the samples could be assigned into 17 phyla. Generally, the bacterial sequences that could be affiliated with known phyla or candidate divisions ranged from 99.93% to 100% in all samples. The dominant high-quality reads could be assigned into 4 phyla, Proteobacteria, Firmicutes, Actinobacteria and Bacteroidetes (accounting for 98.65–99.96% in all samples). In detail (Figure 2a), most of the bacteria in the wetland water samples belonged to Proteobacteria, especially in the September samples (75.76% and 82.00%), while its proportion was only 12.39% in the I sample in May. Firmicutes was the dominant bacterial phylum in May (68.54%) but was third and fourth in June (12.89% and 10.15%) and September (0.51 and 1.04%), respectively. The relative abundances of Actinobacteria were higher in May (18.27%) and June (34.01% and 24.50%) than in September (5.50 and 7.90%). Moreover, microorganisms from the phylum Bacteroidetes accounted for only 0.75% in May. The relative abundances of these four major bacterial phyla illustrated significant temporal variation in the bacterial community composition of the wetland water. In addition, the relative abundance of Proteobacteria was higher in the D zone of the outlet from the wetland to the Liaohe River than in the U zone, while Firmicutes showed the opposite tendency. Compared with wetland water samples, the difference between the U and D water samples could be well attributed to the wetland water input. At the class, order and family levels, notable temporal variations were also found in the wetland water samples. The relative abundances of the U and D water samples showed some differences. For example, Planococcaceae was the most abundant bacterial family and accounted for 58.12% of the total reads in May but only 0.19% and 1.95% in June and 0.16% and 0.27% in September, respectively (Figure 2b). Probably due to the low abundances of Planococcaceae in the water samples in the mature stage of reeds, the abundance of Planococcaceae was lower in the D zone than in the U zone.

Temporal differences in the bacterial community composition among the wetland water samples also existed at the genus level (Table 2). For example, the genera *Psychrobacillus* and *Sporosarcina* had a high proportion in the May water sample but had a much lower abundance in the June and September water samples. As the reeds were in the initial growth stage, the bacterial communities in the wetland water may not have been strongly affected by the reeds. In particular, unclassified bacteria in the family Chesapeake Delaware Bay occurred at a very high proportion (42.78% and 47.57%) in the September water samples. In addition, *Planococcus* showed a much higher abundance in the U and D zones of the outlet from the wetland to the Liaohe River compared with the abundance in the wetland water samples and showed a higher abundance than the other genera in the U and D water samples. *Planococcus* was dominant in Liaohe River and negligibly affected by wetland effluent.

**Figure 3:**
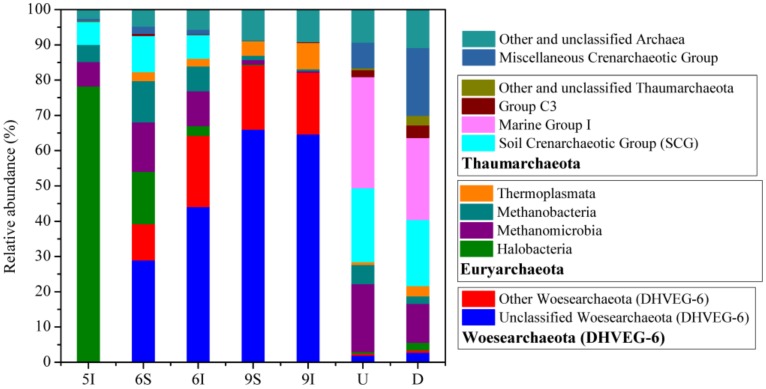
Taxonomic composition of the archaeal communities in the LEW. Rare archaeal populations accounted for <0.5% of the total sequences, and unclassified archaea were included in the group “Other and unclassified Archaea”.

Taxonomic analysis demonstrated that most of the sequences (92.77%) obtained from the LEW samples could be assigned into four archaeal phyla: Woesearchaeota (DHVEG-6) (39.45% of the total reads in all samples), Euryarchaeota (30.64%), Thaumarchaeota (18.29%) and Miscellaneous Crenarchaeotic Group (4.40%) (Figure 3). Generally, there was obvious temporal variation in the water samples, and specifically, the archaeal phylogenetic groups/lineages of the initial growth stage of reeds were significantly different from those of the rapid growth and mature stages of reeds in quantity and type. For example, the relative abundance of Woesearchaeota (DHVEG-6) in May was only 0.01% but increased to 39.30–84.28% in June and September. Furthermore, the proportion of unclassified Woesearchaeota (DHVEG-6) was notably high (75.52% in Woesearchaeota (DHVEG-6) and 50.99% of the total reads in the June and September water samples). The relative abundances of these dominant Woesearchaeota (DHVEG-6) populations increased as the reeds grew, suggesting that these populations may be closely related to the growth of reeds in the wetland (Figure 3). The highest relative abundance of Halobacteria was found in the May wetland water (Figure 3). For the U and D water samples of the outlet from the wetland to the Liaohe River, different with those in wetland water samples, Thaumarchaeota and Euryarchaeota were illustrated to be the dominant phyla, and the frequently detected classes were Marine Group I and Soil Crenarchaeotic Group (SCG) (Figure 3). In the LEW water samples, the high potential for methanogenesis was proven, as demonstrated by the large distributions of classes Methanomicrobia and Methanobacteria; however, the relative abundance of Halobacteria had no direct correlation with SAL (Spearman correlation, *p* > 0.5), which might be controlled by other environmental variables.

**Figure 4:**
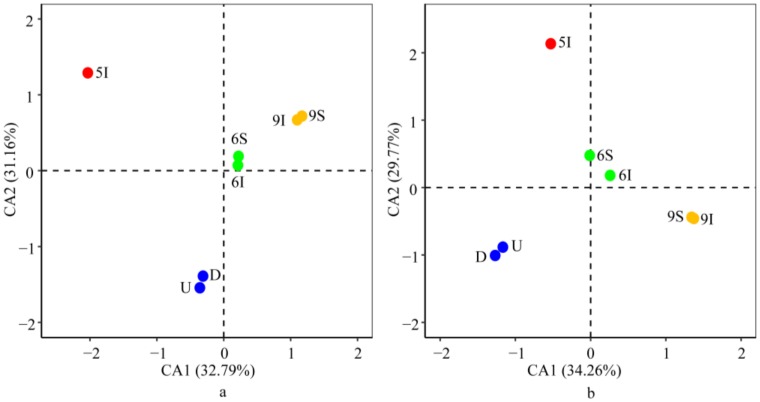
Correspondence analysis (CA) of the bacterial (**a**) and archaeal (**b**) community structures at the OTU level in the LEW.

Consistent with the results of the bacterial and archaeal community taxonomic compositions, ordinal analysis at the OTU level demonstrated that the bacterial and archaeal community structures in the wetland water samples both had evident temporal differences and showed the same characteristics between samples U and D from the Liaohe River (Figure 4). These results suggested that (i) seasonal variation related to the different growth periods of reeds was the main factor influencing the bacterial and archaeal community structures in the wetland; (ii) furthermore, the wetland effluent contributed little to the bacterial and archaeal community structures of the receiver, the Liaohe River. In addition, at the phylum and genus levels, the bacterial and archaeal community distribution patterns were in accordance with those at the OTU level (Figure S1).

### 3.4. Abundances of the Bacterial and Archaeal 16S rRNA Genes

**Figure 5:**
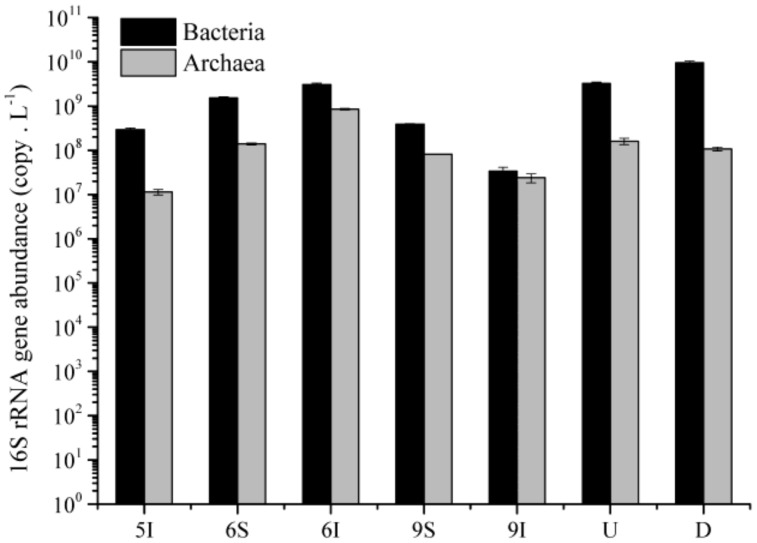
Abundances of the bacterial and archaeal 16S rRNA genes in the water samples from the LEW.

The qPCR results revealed that the abundances of the bacterial and archaeal 16S rRNA genes in the water from the LEW ranged from 3.39 × 10^7^ to 3.09 × 10^9^ copy/L and 1.14 × 10^7^ to 8.54 × 10^8^ copy/L, respectively (Figure 5). Significant temporal differences were observed in the bacterial and archaeal 16S rRNA gene abundances between the samples in June and September (Mann–Whitney test, *p* < 0.05 and *p* < 0.01), and obvious spatial differences were also found between the wetland samples in the S and I zones in June and September (*p* < 0.05). Moreover, the abundances of bacterial and archaeal 16S rRNA genes in the June samples were obviously higher than those in the May and September samples in the I zone (Figure 5), indicating that the rapid growth stage of reeds harbored the highest bacterial and archaeal 16S rRNA gene abundances.

The abundance of bacterial 16S rRNA genes was lower in the U zone than in the D zone of the outlet from the wetland to the Liaohe River, but the result was opposite for the archaea (Figure 5). Statistical analysis indicated that the abundances of bacterial 16S rRNA genes were significantly higher than those of archaea (Wilcoxon test, *p* < 0.05). The relative abundances of bacterial 16S rRNA genes to the total bacterial and archaeal 16S rRNA genes were 74.70% and 96.98% in the wetland and outlet, respectively.

### 3.5. Effect of the Environmental Parameters on the Bacterial and Archaeal Communities

#### 3.5.1. Effect of the Environmental Parameters on the Abundances and α-Diversity Indices of the Bacterial and Archaeal Communities

Spearman’s correlation analysis demonstrated that none of the environmental parameters were significantly correlated with the abundances of the bacterial and archaeal 16S rRNA genes, except that DOC was closely related to the abundance of bacterial 16S rRNA genes (*r* = −0.857, *p* < 0.05). Stepwise regression analysis revealed that for the bacterial communities in the LEW, DOC was negatively associated with the Shannon diversity and Pielou evenness, and DO was most significantly correlated with the Chao 1 richness (Table 3). The Pielou evenness was also greatly affected by T, DIN and SAL, whereas the Shannon diversity was affected by DIN/PO_4_-P (Table 3, *p* < 0.001). Overall, TP was the environmental parameter with the most influence on the α-diversity of the LEW archaeal communities, and DOC was related to the Shannon diversity in the bacterial and archaeal communities (Table 3).

#### 3.5.2. Correlations of the Detection Frequencies of the Dominant Bacterial and Archaeal Taxa with the Environmental Parameters

Details of the direct correlation analysis between the detection frequencies of the dominant taxa (accounting for >0.5% of the total sequences) and the environmental parameters are listed in Tables S4 and S5. Among all the environmental parameters, DO and TP had significant correlations with 27 and 25 bacterial taxa, respectively, while pH and TP had significant correlations with 11 and 7 archaeal taxa, respectively; these parameters were much more influential than the other parameters (Table S6). However, as the environmental factors were autocorrelated (Table S2), partial correlation analysis was used to distinguish the effects of the different parameters. The results indicated that in the water of the LEW, DO had the greatest effect on the dominant bacterial taxa, successively followed by Chla, pH, TP, DOC, total nitrogen (TN) and NH_4_-N, while pH had the greatest effect on the dominant archaeal taxa, followed by TP (Table S6).

In general, the detection frequency of Methylophilales showed a marginally positive correlation with DO (*r* = 0.964, *p* < 0.001) and negative correlation with TP (*r* = −0.786, *p* = 0.036) (Table S4). When autocorrelation between DO and TP was considered, the detection frequency of Methylophilales emerged as having a significant positive correlation with DO after controlling for TP (*r* = 0.970, *p* = 0.001) but had no significant correlation with TP after controlling for DO (*r* = 0.504, *p* > 0.05). However, the correlation of the detection frequency of Proteobacteria with DO and TP became nonsignificant after controlling for each other, implying that the coeffects of these environmental factors may structure the distribution of Proteobacteria in the LEW.

#### 3.5.3. Effect of the Environmental Parameters on the Bacterial and Archaeal Community Structures

**Figure 6:**
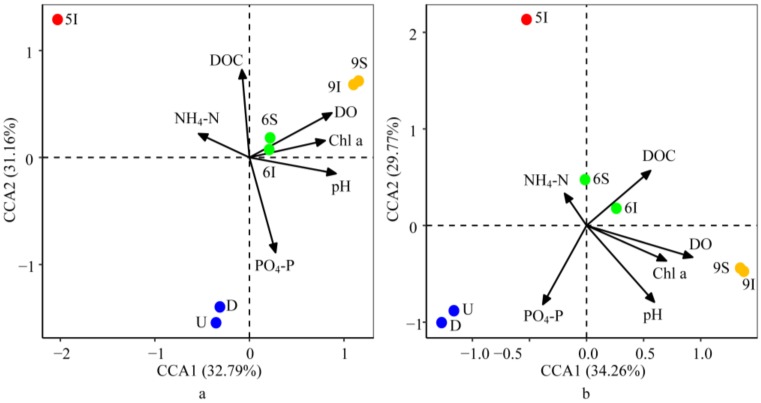
Canonical correspondence analysis (CCA) of the bacterial (**a**) and archaeal (**b**) community structures with the environmental parameters in the LEW.

After considering autocorrelation between the environmental parameters, BIOENV/BVSTEP was applied to select the environmental parameters that affected the bacterial or archaeal community structures the most. The CCA biplot showed that axis 1 and axis 2 explained 32.79% and 31.16% of the variation for the bacteria and 34.26% and 29.77% of the variation for the archaea, respectively. The results indicated that the effects of the environmental parameters on the bacterial and archaeal community structures were generally consistent (Figure 6). DO, PO_4_-P and pH showed highly significant correlations with the bacterial community structures successively, while pH affected the archaeal community structures the most, followed by DO and PO_4_-P, as determined with 999 Monte Carlo permutations (*p* < 0.05). DO and pH mostly impacted the bacterial and archaeal communities in the wetland water samples at the mature stage of reeds, while PO_4_-P mostly impacted the bacterial and archaeal communities in the U and D water samples. Furthermore, consistent with the results of the CCA analysis, the Mantel test demonstrated that DO, pH, PO_4_-P, T and DOC were the essential factors for the bacterial and archaeal community structures (*p* < 0.05).

## 4. Discussion

### 4.1. Distinct Seasonal Dynamics of Planktonic Bacterial and Archaeal Communities in the Wetland

The microbial communities in the sediments, soils and rhizospheres of various wetlands that are associated with different specific characteristics and functions have been explored extensively [2,5,27,28]; however, studies on the microbial communities in wetland waters remain limited [29]. In the current study, we investigated the planktonic microbial community in an artificially irrigated estuarine wetland and its receiving river as well as the responses of the community to environmental parameters.

Our results indicated that the bacterial and archaeal 16S rRNA gene abundances varied considerably in different seasons in the wetland. Considering the different water temperatures in May, June and September, this phenomenon could be due to plant growth characteristics at different stages that affect the physical and chemical characteristics of water. Numerous studies have demonstrated that different vegetative periods can markedly influence the soil, sediment and rhizosphere microbial population abundances in wetlands [30,31,32]; however, few studies have explored the relationship between plant growth stage and microbial population abundance in wetland water, although it has been proven that plants influence planktonic bacterial abundance [33]. It was reported that 50% plant cover was the best ratio to promote the growth of diverse microbial communities and obtain the most efficient wetlands [34], which explains the difference in the bacterial and archaeal abundances in the S and I reed zones in the LEW. Overall, vegetation growth stage and coverage play vital roles in determining wetland planktonic microbial communities.

Temporal and spatial variations in microbial biodiversity have been previously observed in natural wetlands [9,31,35] and constructed wetlands [4,14,30,32], but these studies have mostly emphasized soils [4,9,14,31,32], sediments [9,35] and rhizospheres [30]. The present study revealed the temporal and spatial dynamics of bacterial and archaeal richness, diversity and evenness in the water of an estuarine wetland. The results indicated that the bacterial communities tended to have higher diversity indices in the wetland water in summer, while higher diversity indices for the archaeal communities were found in the wetland water in autumn. In mangrove and intertidal wetland mudflats, summer samples had higher diversity indices than winter samples of bacterial communities, while archaeal communities had lower diversity indices in summer [35]. In addition, the Chao 1 richness, Shannon diversity and Pielou evenness in the wetland water were much lower in the observed planktonic bacterial communities than in the archaeal communities, while the richness and diversity of the bacterial communities were much higher than those of the archaeal communities in the wetland soils [14,32,36,37].

In the wetland, there were seasonal changes in both the bacterial and archaeal community compositions and structures. Previous studies have also reported seasonal variation in bacterial and archaeal community compositions and structures in natural and constructed wetlands [27,32]. Verrucomicrobia, Proteobacteria, Bacteroidetes, Firmicutes and Actinobacteria were reported to be the dominant phyla of the planktonic bacterial communities in wetlands [29,38,39,40,41]. In this study, although their proportions showed seasonal shifts, Proteobacteria, Firmicutes, Actinobacteria and Bacteroidetes were dominant in all samples taken from the LEW. Verrucomicrobia accounted for only 0.09% of all samples, which may be due to the alkaline environment of the LEW, as Verrucomicrobia was found to be closely associated with the most oligotrophic aquatic ecosystems and low pH values [42]. In the soils and sediments of wetlands, Euryarchaeota, Thaumarchaeota, Crenarchaeota, Woesearchaeota and Bathyarchaeota have all been detected as dominant phyla [32,36,37]; however, until now, limited reports have documented the major archaeal groups present in wetland water. In the wetland, the class Halobacteria was dominant in the wetland water in May, which was concordant with previous observations of planktonic archaeal communities [43,44], but different from the U and D water samples. This phenomenon suggests that different habitats might have a significant influence on the archaeal communities. It is worth noting that Woesearchaeota (DHVEG-6) was dominant in the wetland waters in June and September. Woesearchaeota was reported as the major archaeal group in constructed wetland systems planted with *Cyperus papyrus* or *Canna indica L.*, and the metabolic capacities and ecological functions of this group require further study [36].

### 4.2. Negligible Effect of the Wetland Effluent on the Bacterial and Archaeal Communities in the Liaohe River

In the Liaohe River, after receiving water from the wetland, no obvious changes were observed in the bacterial and archaeal community structures between the U and D water samples. The proportions of the dominant bacteria and archaea in the U and D water were obviously different from those in the wetland water samples. In the current study, Proteobacteria, Firmicutes and Actinobacteria were dominant in the bacterial communities and Euryarchaeota and Thaumarchaeota were dominant in the archaeal communities in the U and D samples, which was consistent with the results of other studies in estuarine waters [45,46,47,48]. Therefore, for reed wetland management, the policy of wetland drainage into the Liaohe River every autumn to harvest reeds is feasible and acceptable for the environment of the Liaohe River.

### 4.3. DO and pH as the Most Influential Factors in Shaping the Planktonic Bacterial and Archaeal Communities in the Liaohe River Estuarine Wetland (LEW)

In this study, the abundance of bacterial 16S rRNA genes was significantly negatively correlated with DOC. It was reported that high DOC concentrations stimulated bacterial abundances and productivity in a riverine wetland [49], but bacterial abundances in Lake Namco showed no connection with DOC [50]. This suggested that bacterial abundance could be affected by complex factors. In contrast to the negative relationships between water DOC, NH_4_-N, DIN and TP and the bacterial and archaeal α-diversity indices, a previous study reported that the supply of available nutrients (TOC, TN and TP) positively influenced planktonic microbial diversity [51]. This phenomenon can be explained by the fact that not only the concentration but also the balance of available resources can determine biodiversity [52].

Many studies have proven that cotemporary environmental factors (T, SAL, pH, heavy metals, nutrients and microbial components) as well as environmental characteristics (such as stream width) drive the distribution of planktonic bacterial and archaeal communities [53,54,55]. In this study, DO was the most important factor for the dominant bacterial taxa. The four dominant phyla were all significantly correlated with DO and TP, except Actinobacteria, which was markedly affected by pH and TN. In a high-altitude freshwater wetland [29], Actinobacteria had a significant positive correlation with NO_2_-N, while the other phyla did not show a close correlation with the water chemical parameters. Moreover, pH was the most important factor for the dominant archaeal taxa. Interestingly, pH was also observed to be the most influential factor shaping the archaeal communities in coastal wetland soil [35] and tropical soil [56]. In contrast with the results of saline lake waters, Halobacteria of Euryarchaeota increased with salinity [43]; however, in this wetland system, Halobacteria was not directly correlated with SAL but was markedly negatively correlated with pH. These findings support the traditional perspective that local environmental conditions are important drivers of variation in microbial communities [53].

### 4.4. Potential Roles of the Dominant Bacterial and Archaeal Taxa in the LEW

Microorganisms belonging to the phyla Proteobacteria and Firmicutes are able to degrade a variety of organic compounds, which accounts for the effective removal of DOC [14]. For example, the genera *Pseudomonas*, *Acinetobacter* and *Bacillus* have been linked to the biological degradation of organic and natural compounds [57,58]; however, no significant correlations were observed between these genera and DOC in the LEW, except for the genus *Acinetobacter*. Wetlands play an important role in methane emissions, and the methanogenic microorganisms involved in this process are also assumed to play vital roles in degrading organic carbon [59]. In the LEW, except for the large distributions of archaeal classes Methanomicrobia and Methanobacteria, high abundances of methanogenic microorganisms in the order Methylococcales of Gammaproteobacteria and Methylophilales of Betaproteobacteria were also observed, including the dominant genera *Methanosarcina*, *Methanobacterium* and *Methanosaeta*, suggesting their methanogenic activity and ability to degrade organic compounds. Moreover, the lowest proportion of methanogenic microorganisms was observed in September. In a previous study, the population of methanogenic archaea was significantly smaller in September and October than in June, July and August in a *C. angustifolia* marsh but was highest in September in a *C. lasiocarpa* marsh [60]. Therefore, considering temperature, the growth stage of reeds in the wetland can affect methanogen activity.

The distributions of orders Flavobacteriales and Burkholderiales were dominant in June and September in the wetland; these microorganisms take part in denitrifying activity [61], and their presence revealed the improved denitrification ability in June and September compared to that in May. However, in a wetland treating polluted river water, a lower denitrification rate and denitrifier abundance were observed in summer than in spring and winter [62]. The nitrifying microorganisms Nitrosomonadales, *Candidatus Nitrosopumilus* and *Candidatus Nitrososphaera* promote nitrification by oxidizing ammonia [63,64], and they were also detected in the LEW but in lower numbers than the denitrifying microorganisms. This phenomenon could indicate that the ammonia removal process (nitrification-denitrification cycle) in the LEW was mainly limited by nitrification [5]. Moreover, the desulfuration bacteria (Desulfuromonadales and Desulfovibrionales of Deltaproteobacteria) detected in the water samples was more abundant in September. As *Bacillus* and *Pseudomonas* contain rhizobacteria that can promote plant growth [65,66], their abundances tended to be higher when the reeds were at the initial and rapid growth stages.

## 5. Conclusions

In summary, this study provides a comprehensive view of the abundance, diversity, composition, structure and responses to environmental parameters of the bacterial and archaeal communities in the water of the LEW. In this wetland system, the reeds are irrigated with water from the Liaohe River, and water is discharged into the Liaohe River in autumn every year. The results revealed significant temporal variation in the planktonic microbial community in the wetland. Although changes were observed in microbial abundance and α-diversity, the microbial structure was the same between the S zone and I zone. Generally, drainage increased the microbial abundance and α-diversity but had no significant influence on the planktonic microbial composition and structure in the Liaohe River. In addition, the environmental parameters DO and pH had a strong impact on the LEW planktonic bacterial and archaeal communities, respectively, especially for the dominant taxa and community structure. The wetland exhibited methanogenic activity. Our study supplements knowledge regarding microbial communities in wetland waters and their relationship with environmental parameters.

## Figures and Tables

**Table 1:** α-diversity indices of the water bacterial and archaeal communities in the LEW.

	Sample *	OTUs ^†^	Shannon ^†^	Chao 1 ^†^	Pielou ^†^	Good’s Coverage (%) ^†^
Bacteria	5I	192	3.53	259.00	0.46	99.55
	6S	367	5.66	406.47	0.66	99.43
	6I	330	5.47	493.38	0.65	99.26
	9S	375	4.55	473.06	0.53	99.26
	9I	379	4.46	437.13	0.52	99.44
	U	271	3.17	350.00	0.39	99.41
	D	265	4.11	331.16	0.51	99.48
Archaea	5I	69	3.94	100.00	0.64	99.97
	6S	528	7.02	544.88	0.78	99.89
	6I	754	7.79	778.43	0.81	99.79
	9S	532	7.81	546.53	0.86	99.91
	9I	805	8.14	828.66	0.84	99.84
	U	281	5.02	290.56	0.62	99.94
	D	270	5.90	300.17	0.73	99.87

* The uppercase letters S and I refer to the scarce and intensive reed zones in the wetland, respectively, while U and D refer to the upstream and downstream zones of the outlet from the wetland to the Liaohe River, respectively. The digits indicate the sampling date. ^†^ The reads were normalized to 14,776 for the bacteria and to 27,695 for the archaea.

**Table 2:** Proportion (%) of the bacterial sequences assigned to the top 30 bacterial genera in the total bacterial sequences from each water sample collected from the LEW.

Genera	5I	6S	6I	9S	9I	U	D
*Planococcus*	-	-	1.48	0.15	0.27	61.32	42.24
*Psychrobacter*	7.00	1.01	17.03	-	-	3.47	10.48
*Psychrobacillus*	34.76	-	0.12	-	-	-	0.98
*Bacillus*	8.13	12.36	3.64	0.18	0.30	0.90	1.20
*Sporosarcina*	23.08	-	-	-	-	-	-
*Pseudomonas*	-	4.49	1.46	0.74	0.28	0.98	10.23
*Mycobacterium*	4.95	1.95	1.14	0.28	0.17	5.93	1.98
*CL500-29 marine group*	0.41	4.05	3.09	0.75	1.08	1.48	0.55
*Flavobacterium*	-	2.25	4.31	1.97	1.91	0.53	-
*Altererythrobacter*	-	1.17	0.84	0.24	0.72	0.32	5.74
*hgcI clade*	-	3.48	3.76	0.22	0.64	0.10	-
*Rheinheimera*	-	0.14	-	4.88	1.71	0.17	-
*Acinetobacter*	-	1.56	3.57	-	-	0.36	0.37
*Limnohabitans*	-	2.17	2.95	0.10	0.15	-	-
*Paracoccus*	-	0.36	1.10	-	-	2.67	1.01
*Stenotrophomonas*	-	-	0.29	-	-	2.87	1.80
*Exiguobacterium*	-	-	4.26	-	-	0.35	0.13
*Brevundimonas*	-	1.78	1.10	0.32	0.18	0.39	-
*Lutibacter*	-	-	-	-	3.64	-	-
*NS3a marine group*	-	1.43	1.77	0.12	0.26	-	-
*Owenweeksia*	-	0.46	0.36	1.88	0.73	-	-
*Candidatus Pelagibacter*	-	-	-	-	-	0.79	1.87
*Rhodobacter*	0.22	0.96	0.57	0.30	0.19	0.18	0.13
*BAL58 marine group*	-	0.42	0.76	0.68	0.63	-	-
*MWH-UniP1 aquatic group*	-	0.61	0.96	0.38	0.37	-	-
*GKS98 freshwater group*	1.56	0.24	0.16	-	-	-	-
*OM43 clade*	-	0.44	0.51	0.30	0.55	-	-
*Methylophilus*	-	-	-	0.98	0.88	-	-
*Methylophaga*	-	-	0.24	0.68	0.22	-	0.70
*Citricoccus*	1.26	-	-	-	-	0.17	0.11

The “-” symbol indicates that the proportion of the bacterial sequences in the total bacterial sequences was <0.1%.

**Table 3:** Stepwise regression analysis between the α-diversity indices of the bacterial communities in the LEW water and the environmental parameters.

Community	α-Diversity Index	*R* ^2^	*p*	*n*	Explanatory Variables (β-Weights)
Bacteria	Shannon	0.995	0.002	7	DIN/PO_4_-P (−0.941) ***, DOC (−0.454) *
	Chao 1	0.901	0.01	7	DO (0.641) *, NH_4_-N (−0.547) *
	Pielou	1.000	<0.001	7	T (0.478) ***, DOC (−0.636) ***, DIN (−0.530) ***, SAL (−0.118) ***
Archaea	Shannon	0.938	0.004	7	TP (−1.029) ***, DOC (−0.412) *
	Chao 1	0.749	0.012	7	Chla (0.865) *
	Pielou	0.860	0.003	7	TP (−0.927) **

*** *p* < 0.001; ** *p* < 0.01; * *p* < 0.05.

## Supplementary Materials

Supplementary materials can be found at https://www.mdpi.com/2076-2607/8/2/198/s1. Figure S1: Correspondence analysis (CA) or principal components analysis (PCA) of the bacterial and archaeal community structures in the LEW. Table S1: The physico-chemical parameters of water samples collected from the LEW. Table S2: Spearman’s correlation analysis between the environmental parameters. Table S3: Difference between two environmental categories. Table S4: Spearman’s correlation analysis between the dominant bacterial populations and the environmental parameters in the LEW. Table S5: Spearman’s correlation analysis between the dominant archaeal populations and the environmental parameters in the LEW. Table S6: The numbers of microbial taxa whose detection frequencies were significantly correlated with the environmental parameters.
