# Supplementary material for: Planktonic Bacterial and Archaeal Communities in an Artificially Irrigated Estuarine Wetland: Diversity, Distribution, and Responses to Environmental Parameters
Source: Microorganisms. 2020 Jan 31;8(2):198. doi: 10.3390/microorganisms8020198 (PMC7074933; doi:10.3390/microorganisms8020198)
Supplement: Supplementary file 1 [file microorganisms-08-00198-s001.pdf]

Planktonic Bacterial and Archaeal Communities in an Artificially Irrigated Estuarine Wetland: Diversity, Distribution, and Responses to Environmental Parameters

Mingyue Li [1,2,3], Tiezhu Mi [1,2,3], Zhigang Yu [2,4], Manman Ma [1,2,3] and Yu Zhen [1,2,3,*]

[1] College of Environmental Science and Engineering, Ocean University of China, Qingdao 266100, China; myligoahead@163.com (M.L.); mitiezhu@ouc.edu.cn (T.M.); mamanman1992@163.com (M.M.)
[2] Laboratory for Marine Ecology and Environmental Science, Qingdao National Laboratory for Marine Science and Technology, Qingdao 266071, China; zhigangyu@ouc.edu.cn
[3] Key Laboratory of Marine Environment and Ecology, Ministry of Education, Ocean University of China, Qingdao 266100, China
[4] Key Laboratory of Marine Chemistry Theory and Technology, Ministry of Education/Institute for Advanced Ocean Study, Ocean University of China, Qingdao 266100, China
* Correspondence: zhenyu@ouc.edu.cn; Tel.: +86-532-66781940

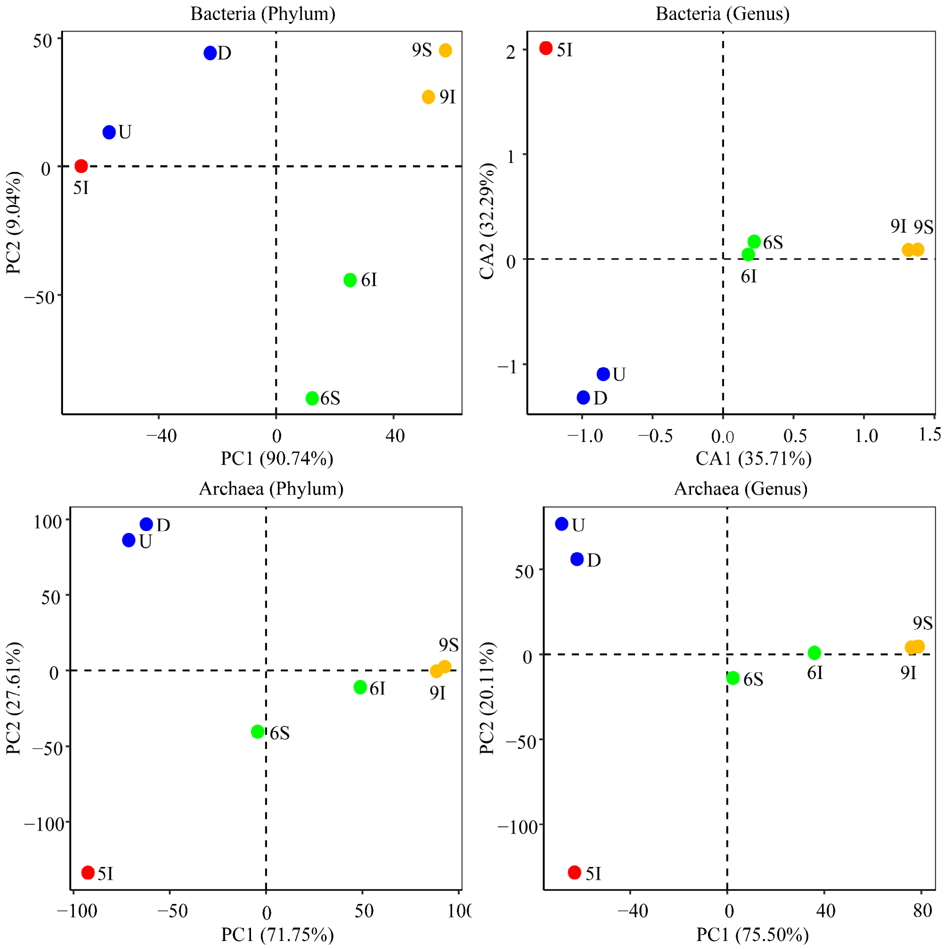

Figure S1 Correspondence analysis (CA) or principal components analysis (PCA) of the bacterial and archaeal community structures in the LEW. "Phylum" is the phylum level, "Genus" is the genus level.

Table S1. The physico-chemical parameters of water samples collected from the LEW (average, n = 4).

Sample	T (℃)	pH	DO (mg/L)	SAL (g/kg)	DOC (mg/L)	TN (mg/L)	DIN (mg/L)	NH_4-N (mg/L)	NO_2-N (mg/L)	NO_3-N (mg/L)	TP (mg/L)	PO_4-P (mg/L)	DIN/PO_4-P	Chla (μg/L)
5I	20.80	7.26	7.58	5.50	15.07	1.97	0.09	0.0519	0.0022	0.0313	0.09	0.01	5.70	0.90
6S	25.45	7.71	8.19	3.98	9.35	1.18	0.01	0.0040	0.0011	0.0000	0.04	0.03	0.16	19.40
6I	25.50	7.83	8.10	3.97	8.97	1.06	0.06	0.0209	0.0014	0.0400	0.05	0.03	2.00	17.58
9S	23.30	8.30	9.14	5.35	13.09	3.33	0.09	0.0236	0.0020	0.0684	0.03	0.03	3.19	16.06
9I	22.43	8.33	9.07	5.35	13.43	2.47	0.07	0.0304	0.0023	0.0333	0.02	0.03	2.08	31.83
U	17.48	7.96	7.80	6.00	9.77	2.56	0.47	0.0143	0.0226	0.4369	0.10	0.06	8.41	15.17
D	18.04	7.88	7.74	18.75	5.32	2.23	0.29	0.0435	0.0139	0.2286	0.09	0.04	6.67	3.63

Abbreviations: temperature (T), dissolved oxygen (DO), salinity (SAL), dissolved organic carbon (DOC), total nitrogen (TN), dissolved inorganic nitrogen (DIN), total phosphorus (TP) and chlorophyll a (Chla).

Table S2. Spearman's correlation analysis between the environmental parameters (n = 7)

	T	pH	DO	SAL	DOC	TN	DIN	NH_4-N	NO_2-N	NO_3-N	TP	PO_4-P	DIN/PO_4-P	Chla
T														
pH	−0.179													
DO	0.536	0.643												
SAL	−0.964***	0.143	−0.571											
DOC	−0.143	0.107	0.107	0.036										
TN	−0.536	0.750	0.357	0.464	0.357									
DIN	−0.857*	0.357	−0.321	0.821*	0.000	0.714								
NH_4-N	−0.357	−0.036	−0.429	0.500	0.357	0.107	0.286							
NO_2-N	−0.929**	0.429	−0.393	0.893**	0.071	0.571	0.857*	0.357						
NO_3-N	−0.571	0.500	−0.107	0.536	−0.357	0.571	0.857*	0.000	0.714					
TP	−0.643	−0.393	−0.821*	0.607	−0.357	−0.071	0.607	0.036	0.536	0.536				
PO_4-P	−0.523	0.450	−0.090	0.487	−0.487	0.234	0.450	−0.288	0.667	0.631	0.414			
DIN/PO_4-P	−0.929**	0.179	−0.536	0.893**	0.071	0.571	0.964***	0.393	0.893**	0.750	0.714	0.414		
Chla	0.607	0.429	0.786*	−0.643	−0.036	−0.107	−0.643	−0.536	−0.429	−0.357	−0.750	0.126	−0.750	

*** $p < 0.001$; ** $p < 0.01$; * $p < 0.05$.

Table S3 Difference between two environmental categories (Mann-Whitney test) (n = 8 except n = 4 for 5)

Groups*	T	pH	DO	SAL	DOC	TN	DIN	NH_4-N	NO_2-N	NO_3-N	TP	PO_4-P	DIN/PO_4-P	Chla
5 vs 6														
5 vs 9		*	*					*			*	*		*
6 vs 9		**	*	**	**						*			
S vs I														
U vs D				*			*	*		*		*		*

* The uppercase letters S and I refer to the scarce and intensive reed zones in the wetland, respectively, while U and D refer to the upstream and downstream zones of the outlet from the wetland to the Liaohe River, respectively. The digits indicate the sampling date. *** $p < 0.001$; ** $p < 0.01$; * $p < 0.05$.

Table S4 Spearman's correlation analysis between the dominant bacterial populations and the environmental parameters in the LEW (phylum and class)

Environmental parameters	Proteo bacteria	Firmicutes	Actino bacteria	Bacteroi detes	Bacilli	Alpha proteobacteria	Actino bacteria	Gamma proteobacteria	Flavo bacteriia	Beta proteobacteria	Acidi microbiia	Delta proteobacteria	Sphingo bacteriia	Cytophagia
T (^{0}C)	0.607	−0.607	0.357	0.536	−0.607	0.536	0.357	0.214	0.607	0.964***	0.429	0.679	0.500	0.571
pH	0.643	−0.643	−0.821*	0.714	−0.643	0.643	−0.821*	−0.143	0.607	−0.143	−0.071	0.357	0.679	0.607
DO (mg/L)	0.929**	−0.929**	−0.393	0.929**	−0.929**	0.929**	−0.393	−0.071	0.821*	0.571	0.250	0.857*	0.964***	0.964***
SAL (g/kg)	−0.571	0.571	−0.393	−0.571	0.571	−0.500	−0.393	−0.071	−0.643	−1.000***	−0.500	−0.643	−0.536	−0.536
DOC (mg/L)	−0.036	0.036	−0.286	0.107	0.036	0.036	−0.286	−0.857*	0.000	−0.036	−0.536	0.321	0.143	0.000
TN (mg/L)	0.250	−0.250	−0.857*	0.250	−0.250	0.214	−0.857*	−0.214	0.036	−0.464	−0.464	0.143	0.286	0.286
DIN (mg/L)	−0.321	0.321	−0.571	−0.357	0.321	−0.429	−0.571	0.107	−0.464	−0.821*	−0.500	−0.536	−0.393	−0.357
NH_4-N (mg/L)	−0.214	0.214	−0.429	−0.321	0.214	−0.250	−0.429	−0.107	−0.357	−0.500	−0.857*	−0.071	−0.393	−0.286
NO_2-N (mg/L)	−0.393	0.393	−0.500	−0.286	0.393	−0.357	−0.500	−0.179	−0.321	−0.893**	−0.357	−0.607	−0.321	−0.429
NO_3-N (mg/L)	−0.036	0.036	−0.500	−0.071	0.036	−0.214	−0.500	0.393	−0.107	−0.536	−0.143	−0.464	−0.179	−0.143
TP (mg/L)	−0.821*	0.821*	0.286	−0.821*	0.821*	−0.893**	0.286	0.286	−0.750	−0.607	−0.036	−0.964***	−0.857*	−0.857*
PO_4-P (mg/L)	−0.126	0.126	−0.126	0.018	0.126	−0.018	−0.126	0.108	0.072	−0.487	0.414	−0.523	0.018	−0.126
DIN/PO_4-P	−0.536	0.536	−0.429	−0.536	0.536	−0.607	−0.429	0.000	−0.607	−0.893**	−0.536	−0.679	−0.571	−0.571
Chla (μg/L)	0.714	−0.714	0.036	0.857*	−0.714	0.857*	0.036	−0.214	0.893**	0.643	0.643	0.643	0.893**	0.750

Table S4 -continued (order)

Environmental parameters	Bacillales	SAR11 clade	Pseudomonadales	Micrococcales	Flavobacteriales	Corynebacteriales	Rhodobacterales	Acidimicrobiales	Burkholderiales	Sphingomonadales
T (^{0}C)	−0.607	0.571	0.286	0.821*	0.607	−0.500	0.286	0.429	0.929**	0.321
pH	−0.643	0.679	−0.679	−0.571	0.607	−0.607	−0.607	−0.071	−0.357	0.000
DO (mg/L)	−0.929**	0.893**	−0.500	0.143	0.821*	−0.714	−0.321	0.250	0.393	0.143
SAL (g/kg)	0.571	−0.536	−0.179	−0.786*	−0.643	0.393	−0.250	−0.500	−0.964**	−0.143
DOC (mg/L)	0.036	0.000	−0.750	0.036	0.000	−0.036	−0.714	−0.536	0.000	−0.821*
TN (mg/L)	−0.250	0.179	−0.786*	−0.786*	0.036	−0.143	−0.786*	−0.464	−0.536	−0.393
DIN (mg/L)	0.321	−0.393	−0.250	−0.964***	−0.464	0.321	−0.429	−0.500	−0.786*	−0.321
NH_4-N (mg/L)	0.214	−0.179	−0.107	−0.214	−0.357	−0.179	−0.500	−0.857*	−0.429	−0.214
NO_2-N (mg/L)	0.393	−0.321	−0.321	−0.893**	−0.321	0.250	−0.357	−0.357	−0.929**	−0.250
NO_3-N (mg/L)	0.036	−0.107	−0.036	−0.893**	−0.107	0.107	−0.214	−0.143	−0.571	−0.036
TP (mg/L)	0.821*	−0.857*	0.464	−0.464	−0.750	0.821*	0.357	−0.036	−0.464	−0.107
PO_4-P (mg/L)	0.126	−0.018	0.000	−0.595	0.072	0.144	0.234	0.414	−0.631	0.342
DIN/PO_4-P	0.536	−0.571	−0.179	−0.893**	−0.607	0.464	−0.357	−0.536	−0.821*	−0.393
Chla (μg/L)	−0.714	0.821*	−0.250	0.429	0.893**	−0.607	0.107	0.643	0.429	0.357

Table S4 -continued (order)

Environmental parameters	Frankiales	PeM15	Desulfuromonadales	Xanthomonadales	Rhizobiales	Sphingobacteriales	Chromatiales	Caulobacterales	Methylophilales
T (^{0}C)	0.857*	−0.464	0.178	−0.464	−0.107	0.500	0.036	0.571	0.571
pH	−0.286	−0.429	0.757*	0.536	−0.500	0.679	0.643	−0.036	0.679
DO (mg/L)	0.321	−0.571	0.802*	0.036	−0.357	0.964***	0.750	0.464	0.964***
SAL (g/kg)	−0.893**	0.393	−0.178	0.429	0.071	−0.536	−0.179	−0.679	−0.607
DOC (mg/L)	0.071	−0.357	0.445	−0.500	−0.607	0.143	0.607	−0.429	0.071
TN (mg/L)	−0.679	−0.214	0.668	0.536	−0.357	0.286	0.714	−0.286	0.321
DIN (mg/L)	−0.929**	0.214	0.045	0.750	0.000	−0.393	0.071	−0.464	−0.286
NH_4-N (mg/L)	−0.357	−0.393	0.134	−0.214	−0.607	−0.393	−0.250	−0.964***	−0.357
NO_2-N (mg/L)	−0.786*	0.286	−0.045	0.607	−0.071	−0.321	0.000	−0.536	−0.357
NO_3-N (mg/L)	−0.714	0.143	0.045	0.964***	0.071	−0.179	0.000	−0.107	0.000
TP (mg/L)	−0.536	0.750	−0.757*	0.464	0.607	−0.857*	−0.571	−0.143	−0.786*
PO_4-P (mg/L)	−0.414	0.523	−0.225	0.721	0.378	0.018	−0.108	0.162	−0.090
DIN/PO_4-P	−0.893**	0.321	−0.134	0.607	0.036	−0.571	−0.071	−0.571	−0.500
Chla (μg/L)	0.643	−0.286	0.401	−0.179	−0.143	0.893**	0.429	0.607	0.750

Table S4 -continued (family)

Environmental parameters	Planococcaceae	Chesapeake Delaware Bay	Moraxellaceae	Microbacteriaceae	Bacillaceae	Rhodobacteraceae	Flavobacteriaceae	Pseudomonadaceae	Mycobacteriaceae	Acidimicrobiaceae
T (^{0}C)	−0.714	0.571	0.000	0.821*	0.250	0.286	0.750	0.107	−0.643	0.429
pH	−0.286	0.679	−0.607	−0.571	−0.929**	−0.607	0.321	−0.143	−0.536	−0.071
DO (mg/L)	−0.857*	0.893**	−0.714	0.143	−0.536	−0.321	0.679	−0.071	−0.821*	0.250
SAL (g/kg)	0.643	−0.536	0.071	−0.786*	−0.214	−0.250	−0.786*	0.000	0.571	−0.500
DOC (mg/L)	0.000	0.000	−0.571	0.036	−0.214	−0.714	−0.214	−0.964***	−0.107	−0.536
TN (mg/L)	−0.071	0.179	−0.607	−0.786*	−0.857*	−0.786*	−0.286	−0.321	−0.071	−0.464
DIN (mg/L)	0.536	−0.393	0.071	−0.964***	−0.536	−0.429	−0.643	−0.036	0.500	−0.500
NH_4-N (mg/L)	0.250	−0.179	0.143	−0.214	−0.143	−0.500	−0.536	−0.429	0.000	−0.857*
NO_2-N (mg/L)	0.679	−0.321	0.000	−0.893**	−0.464	−0.357	−0.500	−0.107	0.429	−0.357
NO_3-N (mg/L)	0.393	−0.107	0.214	−0.893**	−0.607	−0.214	−0.214	0.250	0.286	−0.143
TP (mg/L)	0.857*	−0.857*	0.679	−0.464	0.286	0.357	−0.607	0.321	0.929**	−0.036
PO_4-P (mg/L)	0.396	−0.018	0.036	−0.595	−0.252	0.234	0.072	0.487	0.234	0.414
DIN/PO_4-P	0.714	−0.571	0.179	−0.893**	−0.357	−0.357	−0.750	−0.107	0.643	−0.536
Chla (μg/L)	−0.643	0.821*	−0.536	0.429	−0.143	0.107	0.893**	0.071	−0.750	0.643

Table S4 -continued (family)

Environmental parameters	Sporichthy aceae	Comamonad aceae	Erythrobacter aceae	Chromati aceae	Xanthomonad aceae	GR WP33 58	Cryomorph aceae	Methylophil aceae	Caulobacter aceae	Family XII	Alcaligen aceae	Saprospir aceae
T (^{0}C)	0.821*	0.893**	0.179	0.143	−0.571	0.178	0.571	0.571	0.571	0.037	0.607	0.536
pH	0.143	0.000	−0.036	0.821*	0.679	0.757*	0.607	0.679	−0.036	−0.185	−0.643	0.643
DO (mg/L)	0.643	0.643	0.000	0.893**	0.107	0.802*	0.964***	0.964***	0.464	−0.296	−0.071	1.000***
SAL (g/kg)	−0.857*	−0.964***	−0.036	−0.250	0.536	−0.178	−0.536	−0.607	−0.679	−0.074	−0.643	−0.571
DOC (mg/L)	−0.321	−0.143	−0.857*	0.214	−0.357	0.445	0.000	0.071	−0.429	−0.778*	0.393	0.107
TN (mg/L)	−0.393	−0.357	−0.429	0.679	0.679	0.668	0.286	0.321	−0.286	−0.408	−0.571	0.357
DIN (mg/L)	−0.714	−0.714	−0.250	0.071	0.786*	0.045	−0.357	−0.286	−0.464	0.074	−0.643	−0.321
NH_4-N (mg/L)	−0.679	−0.679	−0.286	−0.429	−0.143	0.134	−0.286	−0.357	−0.964***	−0.482	0.036	−0.429
NO_2-N (mg/L)	−0.643	−0.786*	−0.107	0.000	0.714	−0.045	−0.429	−0.357	−0.536	0.074	−0.714	−0.393
NO_3-N (mg/L)	−0.286	−0.357	0.036	0.214	0.929**	0.045	−0.143	0.000	−0.107	0.445	−0.714	−0.107
TP (mg/L)	−0.536	−0.536	0.071	−0.571	0.357	−0.757*	−0.857*	−0.786*	−0.143	0.593	−0.250	−0.821*
PO_4-P (mg/L)	0.018	−0.270	0.523	0.162	0.775*	−0.225	−0.126	−0.090	0.162	0.542	−0.847*	−0.090
DIN/PO_4-P	−0.821*	−0.821*	−0.286	−0.143	0.643	−0.134	−0.571	−0.500	−0.571	0.074	−0.536	−0.536
Chla (μg/L)	0.857*	0.714	0.321	0.607	−0.107	0.401	0.750	0.750	0.607	−0.037	0.000	0.786*

Table S4 -continued (genus)

Environmental parameters	*Planococcus*	*Psychrobacter*	*Psychrobacillus*	*Bacillus*	*Sporosarcina*	*Pseudomonas*	*Mycobacterium*	*CL500−29 marine group*	*Flavobacterium*	*Altererythrobacter*
T (^{0}C)	−0.393	−0.107	−0.357	0.214	−0.582	0.000	−0.571	0.536	0.857*	0.179
pH	0.429	−0.500	−0.679	−0.893**	−0.691	−0.036	−0.607	0.000	0.107	−0.036
DO (mg/L)	−0.143	−0.679	−0.857*	−0.500	−0.946***	0.036	−0.893**	0.429	0.679	0.000
SAL (g/kg)	0.429	0.036	0.286	−0.250	0.582	−0.107	0.643	−0.643	−0.893**	−0.036
DOC (mg/L)	−0.679	−0.321	0.000	−0.107	−0.036	−0.643	−0.321	−0.393	−0.357	−0.857*
TN (mg/L)	0.179	−0.536	−0.321	−0.786*	−0.255	−0.107	−0.214	−0.357	−0.321	−0.429
DIN (mg/L)	0.536	0.071	0.286	−0.500	0.346	0.071	0.429	−0.536	−0.643	−0.250
NH_4-N (mg/L)	−0.071	−0.107	0.143	−0.286	0.273	−0.857*	0.286	−0.929**	−0.714	−0.286
NO_2-N (mg/L)	0.607	0.143	0.214	−0.429	0.346	0.000	0.357	−0.464	−0.714	−0.107
NO_3-N (mg/L)	0.821*	0.214	0.143	−0.571	0.055	0.357	0.214	−0.179	−0.214	0.036
TP (mg/L)	0.464	0.714	0.821*	0.321	0.837*	0.429	0.857*	−0.179	−0.500	0.071
PO_4-P (mg/L)	0.847*	0.216	−0.072	−0.216	0.055	0.595	0.162	0.270	−0.090	0.523
DIN/PO_4-P	0.464	0.214	0.464	−0.321	0.546	0.000	0.571	−0.607	−0.786*	−0.286
Chla (μg/L)	−0.071	−0.357	−0.750	−0.107	−0.800*	0.179	−0.821*	0.714	0.750	0.321

Table S4 -continued (genus)

Environmental parameters	*hgcI clade*	*Rheinheimera*	*Acinetobacter*	*Limnohabitans*	*Paracocccus*	*Stenotrophomonas*	*Exiguobacterium*	*Brevundimonas*	*Lutibacter*	*NS3a marine group*
T (^{0}C)	0.821*	0.143	0.450	0.929**	−0.143	−0.371	0.037	0.571	−0.158	0.857*
pH	0.143	0.821*	−0.324	−0.107	0.107	−0.074	−0.185	−0.036	0.906**	0.107
DO (mg/L)	0.643	0.893**	−0.018	0.536	−0.143	−0.408	−0.296	0.464	0.670	0.607
SAL (g/kg)	−0.857*	−0.250	−0.414	−0.964**	0.071	0.334	−0.074	−0.679	0.059	−0.821*
DOC (mg/L)	−0.321	0.214	−0.883**	0.000	−0.714	−0.741	−0.778*	−0.429	0.493	−0.429
TN (mg/L)	−0.393	0.679	−0.577	−0.536	−0.143	−0.111	−0.408	−0.286	0.788*	−0.464
DIN (mg/L)	−0.714	0.071	−0.306	−0.893**	0.214	0.408	0.074	−0.464	0.256	−0.750
NH_4-N (mg/L)	−0.679	−0.429	−0.613	−0.464	−0.571	−0.408	−0.482	−0.964**	−0.020	−0.536
NO_2-N (mg/L)	−0.643	0.000	−0.414	−0.821*	0.286	0.408	0.074	−0.536	0.335	−0.679
NO_3-N (mg/L)	−0.286	0.214	0.090	−0.607	0.571	0.630	0.445	−0.107	0.256	−0.321
TP (mg/L)	−0.536	−0.571	0.252	−0.643	0.536	0.778*	0.593	−0.143	−0.512	−0.571
PO_4-P (mg/L)	0.018	0.162	0.236	−0.378	0.793*	0.767*	0.542	0.162	0.199	−0.018
DIN/PO_4-P	−0.821*	−0.143	−0.360	−0.929**	0.179	0.408	0.074	−0.571	0.118	−0.857*
Chla (μg/L)	0.857*	0.607	0.180	0.750	0.107	−0.222	−0.037	0.607	0.433	0.821*

*** $p < 0.001$; ** $p < 0.01$; * $p < 0.05$.

Table S5 Spearman's correlation analysis between the dominant archaeal populations and the environmental parameters in the LEW (phylum and class)

Environmental parameters	Woesearchaeota (DHVEG−6)	Euryar chaeota	Thaumar chaeota	Miscellaneous Crenarchaeotic Group	Halobacteria	Methanomicrobia	Methanobacteria	Thermoplasmata	Soil Crenarchaeotic Group (SCG)	Marine Group I	Group C3
T (^{0}C)	0.607	−0.107	−0.429	−0.393	0.179	−0.286	0.321	0.214	−0.429	−0.342	−0.321
pH	0.643	−0.857*	−0.321	−0.357	−1.000***	−0.393	−0.714	0.750	−0.321	0.090	0.071
DO (mg/L)	0.929**	−0.679	−0.536	−0.571	−0.643	−0.429	−0.286	0.750	−0.536	−0.162	−0.214
SAL (g/kg)	−0.571	0.036	0.393	0.429	−0.143	0.214	−0.393	−0.107	0.393	0.306	0.357
DOC (mg/L)	−0.036	0.107	−0.643	−0.750	−0.107	−0.571	−0.429	−0.071	−0.643	−0.739	−0.821*
TN (mg/L)	0.250	−0.571	−0.143	−0.214	−0.750	−0.214	−0.679	0.393	−0.143	0.036	−0.071
DIN (mg/L)	−0.321	−0.214	0.393	0.357	−0.357	0.214	−0.393	−0.107	0.393	0.360	0.286
NH_4-N (mg/L)	−0.214	−0.107	−0.393	−0.250	0.036	−0.571	−0.643	0.036	−0.393	−0.559	−0.393
NO_2-N (mg/L)	−0.393	−0.143	0.321	0.286	−0.429	0.143	−0.464	−0.036	0.321	0.360	0.357
NO_3-N (mg/L)	−0.036	−0.429	0.464	0.429	−0.500	0.286	−0.250	0.036	0.464	0.577	0.500
TP (mg/L)	−0.821*	0.500	0.857*	0.821*	0.393	0.750	0.393	−0.714	0.857*	0.595	0.536
PO_4-P (mg/L)	−0.126	−0.180	0.595	0.559	−0.450	0.505	−0.018	0.162	0.595	0.836*	0.847*
DIN/PO_4-P	−0.536	0.000	0.429	0.393	−0.179	0.250	−0.321	−0.286	0.429	0.306	0.250
Chla (μg/L)	0.714	−0.357	−0.393	−0.429	−0.429	−0.250	0.000	0.607	−0.393	−0.018	0.000

Table S5 - continued (order and family)

Environmental parameters	Halo bacteriales	Methanosarcinales	Methanocellales	Methano bacteriales	20a 9	Thermoplasmatales	Halo bacteriaceae	Methanosarcinaceae	Methanosaetaceae	Methanocellaceae	Methano bacteriaceae
T (^{0}C)	0.179	−0.286	0.393	0.321	0.500	−0.324	0.179	−0.429	0.357	0.393	0.321
pH	−1.000***	−0.393	−0.857*	−0.714	0.679	0.523	−1.000***	−0.321	−0.179	−0.857*	−0.714
DO (mg/L)	−0.643	−0.429	−0.286	−0.286	0.964***	0.000	−0.643	−0.536	0.107	−0.286	−0.286
SAL (g/kg)	−0.143	0.214	−0.429	−0.393	−0.536	0.360	−0.143	0.393	−0.393	−0.429	−0.393
DOC (mg/L)	−0.107	−0.571	0.179	−0.429	0.143	−0.324	−0.107	−0.643	−0.714	0.179	−0.429
TN (mg/L)	−0.750	−0.214	−0.536	−0.679	0.286	0.072	−0.750	−0.143	−0.500	−0.536	−0.679
DIN (mg/L)	−0.357	0.214	−0.429	−0.393	−0.393	0.162	−0.357	0.393	−0.393	−0.429	−0.393
NH_4-N (mg/L)	0.036	−0.571	−0.107	−0.643	−0.393	0.018	0.036	−0.393	−0.821*	−0.107	−0.643
NO_2-N (mg/L)	−0.429	0.143	−0.643	−0.464	−0.321	0.559	−0.429	0.321	−0.321	−0.643	−0.464
NO_3-N (mg/L)	−0.500	0.286	−0.571	−0.250	−0.179	0.342	−0.500	0.464	−0.036	−0.571	−0.250
TP (mg/L)	0.393	0.750	0.107	0.393	−0.857*	0.054	0.393	0.857*	0.143	0.107	0.393
PO_4-P (mg/L)	−0.450	0.505	−0.739	−0.018	0.018	0.836*	−0.450	0.595	0.450	−0.739	−0.018
DIN/PO_4-P	−0.179	0.250	−0.321	−0.321	−0.571	0.162	−0.179	0.429	−0.429	−0.321	−0.321
Chla (μg/L)	−0.429	−0.250	−0.286	0.000	0.893**	0.342	−0.429	−0.393	0.464	−0.286	0.000

Table S5 -continued (genus)

Environmental parameters	*Methanosarcina*	*Halogranum*	*Methanobacterium*	*Halomarina*	*Rice Cluster I*	*Halococcus*	*Halobaculum*	*Methanosaeta*
T (^{0}C)	−0.429	0.143	0.321	0.378	0.393	0.111	0.126	0.357
pH	−0.321	−0.929**	−0.714	−0.937**	−0.857*	−0.927**	−0.955**	−0.179
DO (mg/L)	−0.536	−0.571	−0.286	−0.450	−0.286	−0.630	−0.631	0.107
SAL (g/kg)	0.393	−0.036	−0.393	−0.288	−0.429	0.000	−0.162	−0.393
DOC (mg/L)	−0.643	−0.107	−0.429	−0.036	0.179	−0.074	0.018	−0.714
TN (mg/L)	−0.143	−0.607	−0.679	−0.739	−0.536	−0.704	−0.721	−0.500
DIN (mg/L)	0.393	−0.286	−0.393	−0.505	−0.429	−0.371	−0.360	−0.393
NH_4-N (mg/L)	−0.393	0.214	−0.643	0.162	−0.107	0.259	−0.090	−0.821*
NO_2-N (mg/L)	0.321	−0.429	−0.464	−0.613	−0.643	−0.371	−0.360	−0.321
NO_3-N (mg/L)	0.464	−0.500	−0.250	−0.649	−0.571	−0.593	−0.505	−0.036
TP (mg/L)	0.857*	0.286	0.393	0.108	0.107	0.259	0.414	0.143
PO_4-P (mg/L)	0.595	−0.541	−0.018	−0.673	−0.739	−0.449	−0.364	0.450
DIN/PO_4-P	0.429	−0.143	−0.321	−0.360	−0.321	−0.185	−0.162	−0.429
Chla (μg/L)	−0.393	−0.500	0.000	−0.324	−0.286	−0.408	−0.342	0.464

*** $p < 0.001$; ** $p < 0.01$; * $p < 0.05$.

Table S6 The numbers of microbial taxa whose detection frequencies were significantly correlated with the environmental parameters

Environmental parameters	Number I*	Number II†	Number III‡	Number IV§	Correlative environmental parameters
T (^{0}C)	11	2	0	0	SAL, DIN, NO_2-N, DIN/PO_4-P
pH	9	9	11	11	
DO (mg/L)	27	17	2	1	TP
SAL (g/kg)	12	1	0	0	T, DIN, NO_2-N, DIN/PO_4-P
DOC (mg/L)	8	8	1	1	
TN (mg/L)	8	8	0	0	
DIN (mg/L)	7	2	0	0	T, SAL, NO_2-N, NO_3-N, DIN/PO_4-P
NH_4-N (mg/L)	8	8	2	2	
NO_2-N (mg/L)	7	2	0	0	T, SAL, DIN, DIN/PO_4-P
NO_3-N (mg/L)	5	2	0	0	DIN
TP (mg/L)	25	9	7	6	DO
PO_4-P (mg/L)	5	5	3	3	
DIN/PO_4-P	11	2	0	0	T, SAL, DIN, NO_2-N
Chla (μg/L)	15	15	1	1	

[*] The number of bacterial taxa whose detection frequencies were significantly correlated with the environmental parameters; [†] The number of bacterial taxa whose detection frequencies were significantly correlated with the environmental parameters after controlling the autocorrelation between the environmental parameters; [‡] The number of archaeal taxa whose detection frequencies were significantly correlated with the environmental parameters; [§] The number of archaeal taxa whose detection frequencies were significantly correlated with the environmental parameters after controlling the autocorrelation between the environmental parameters.
